# L'endométriose pariétale cicatricielle après césarienne: une entité rare

**DOI:** 10.11604/pamj.2016.24.79.8680

**Published:** 2016-05-25

**Authors:** Mohammed El Fahssi, Massama Lomdo, Ahmed Bounaim, Abdelmounaim Ait Ali, Khalid Sair

**Affiliations:** 1 Service de Chirurgie Viscérale I de l'Hôpital Militaire Mohammed V, Rabat, Maroc

**Keywords:** Endométriose, paroi abdominale, pariétoplastie, Endometriosis, abdominal wall, parietoplasty

## Abstract

L'endométriose de la paroi est une entité clinique rare, dont la physiopathologie demeure imprécise. Elle survient le plus souvent après une intervention chirurgicale gynécologique ou obstétricale. Nous rapportons le cas d'une patiente présentant une douleur cyclique, au niveau de la cicatrice de césarienne, Avec à l'examen clinique une masse de 5cm localisée au niveau de la fosse iliaque droite. la tomodensitométrie montre une masse de densité tissulaire de 45mm de grand axe. D'où la décision d'excision large de la lésion dont l’étude anatomopathologique confirme le diagnostic d'endométriose pariétale. Les suites postopératoires étaient simples avec un recul de 20 mois sans récidive de la masse ni de la douleur. A travers notre cas, nous insisterons sur les caractéristiques de cette pathologie, ce qui permettra au praticien de comprendre l'intérêt du diagnostic et de la prise en charge précoce de cette affection ainsi que la possibilité de sa prévention au cours de chaque chirurgie gynécologique ou obstétricale.

## Abstract (en)

Wall endometriosis is a rare clinical entity whose pathophysiology remains unclear. It occurs most frequently after gynecologic or obstetric surgery. We report the case of a patient with cyclic pain at the caesarean section scar. Clinical examination showed a 5 cm mass in the right iliac fossa. Tomodensitometry revealed a tissue density mass (45mm on the major axis). Hence, the decision to perform a wide excision of the lesion. Anatomo-pathological examination confirmed the diagnosis of parietal endometriosis. Postoperative sequelae were simple with a follow-up period of 20 months with no recurrence of the mass or of the pain. Our study highlights the characteristics of this disease to allow the health practitioner to understand the importance of diagnosis, of early treatment of this disease as well as of the possibility to prevent it during each gynecologic or obstetric surgery.

## Introduction

L'endométriose se décrit par la présence de tissu endométrial fonctionnel en dehors de la cavité utérine [1]. Sa localisation la plus fréquente siège au niveau des organes génitaux internes. Les autres localisations extra-génitales sont moins fréquentes. Sa survenue au niveau des cicatrices de chirurgie gynécologique ou obstétricale est rare (0,03-0,4%) [2]. Nous rapportons le cas d'une patiente ayant une endométriose de la paroi abdominale sur cicatrice de Pfannenstiel. Ce cas est rapporté en raison de sa rareté et du caractère inhabituel de sa localisation.

## Patient et observation

**Figure 1:**
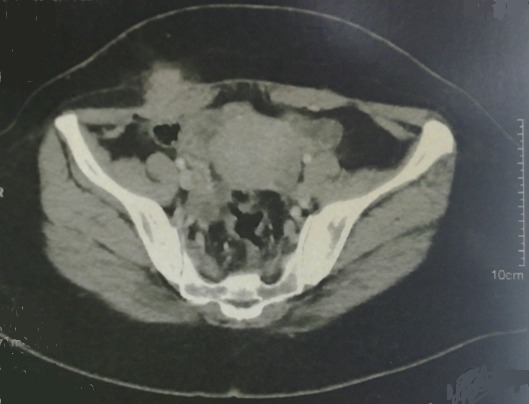
Coupe scannographique montrant la masse en contact avec le muscle grand droit de l'abdomen

**Figure 2:**
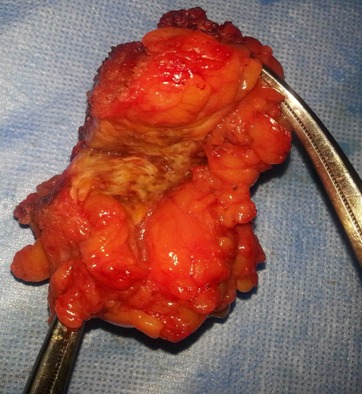
Pièce opératoire d'endométriome ouvert avec piqueté hémorragique

**Figure 3:**
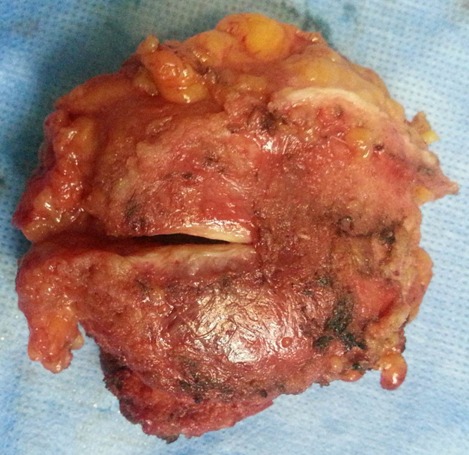
Pièce opératoire d'endométriome montrant la face musculaire

Il s'agit d'une Patiente de 36 ans, deuxième geste deuxième parité, deux enfants vivants ayant bénéficié à deux reprises d'une césarienne, la dernière remontant à trois ans qui se plaint de douleurs au niveau de la cicatrice de césarienne avec développement d'une masse augmentant de taille associée à des douleurs rythmées par le cycle menstruel. L'examen abdominal met en évidence une masse de 5cm de grand axe fixée au plan profond siégeant en regard de l'extrémité droite de la cicatrice de pfannenstiel. La tomodensitométrie montre une masse de densité tissulaire non rehaussée par le produit de contraste mesurant 30 x 45 mm prenant contact intimement avec le muscle droit de l'abdomen, évoquant une tumeur desmoide (Figure 1), dans ce sens Une excision large de la masse a été réalisée (Figure 2 et Figure 3). Devant l'importance de la perte de substance pariétale une pariétoplastie a été réalisée par interposition d'une plaque de Polypropylène 15x15 cm entre les deux plans musculaires profond constitué par le muscle oblique interne et le transverse et le plan superficiel constitué par l'oblique externe. L'examen anatomopathologique de la pièce de résection révélait qu'il s'agit d'un foyer d'endométriose de la paroi abdominale. Les suites postopératoires immédiates ont été simples avec une reprise de l'alimentation le lendemain et une sortie sous traitement antalgique. La patiente a rapporté une disparition des dysménorrhées dans les semaines suivant l'intervention chirurgicale. Un contrôle à 3 mois puis tous les 6 mois a été réalisé n'objectivant aucune récidive sur un recul de 20 mois.

## Discussion

L'endométriose correspond à l'implantation ectopique de tissu endométrial. Sa localisation abdominale a été identifiée au niveau de différents sites dont les muscles grands droits, l'ombilic ainsi que les orifices de trocarts [3]. L'endométriose pariétale cicatricielle est une entité assez rare dont le diagnostic initial n'est pas aisé. Son mécanisme physiopathologique est expliqué par une greffe de cellules endométriales lors de l'intervention chirurgicale favorisée par les œstrogènes produisant ainsi des endométriomes [4]. Les modes de révélation les plus fréquents sont la découverte d'une masse palpable augmentant de volume, douloureuse pouvant être associée à des modifications cutanées en regard de la cicatrice. Le caractère cataménial c'est-à-dire l'exacerbation de ces signes pendant les règles est un élément important du diagnostic. Il a été retrouvé chez notre malade. L’échographie confirme l'origine pariétale typiquement intramusculaire de la masse retrouvée cliniquement, le scanner et la résonance magnétique nucléaire permettent d'orienter le diagnostic sans pour autant donner de certitude car seule l'anatomopathologie permet la confirmation. Le traitement de ces lésions repose sur une exérèse chirurgicale, celle-ci doit être aussi large que possible afin d'enlever toute la masse. Cette chirurgie peut être délabrante nécessitant une reconstruction pariétale avec pariétoplastie ce qui a été le cas chez notre malade. La prévention en cas de laparotomie est basée sur le lavage abondant de la cavité abdominale et de la cicatrice en fin d'intervention ainsi que le changement de gants pour le temps de fermeture pariétale, alors qu'en cœlioscopie, l'extraction des pièces opératoires dans un sac de protection et le lavage abondant de la cavité pelvienne devraient être systématiques. Ainsi, ces mesures relèvent de la bonne pratique chirurgicale bien que leur bénéfice n'a jamais été démontré [3].

## Conclusion

L'endométriose cicatricielle pariétale est une pathologie certes rare mais dont le diagnostic doit être évoqué notamment chez des femmes ayant bénéficié de chirurgie gynécologique ou obstétricale à ciel ouvert et présentant des douleurs associées ou non à des troubles menstruels. Le traitement est essentiellement chirurgical.
